# Migraine: An Underestimated Neurological Condition Affecting Billions

**DOI:** 10.7759/cureus.28347

**Published:** 2022-08-24

**Authors:** Jatin Gupta, Sagar S Gaurkar

**Affiliations:** 1 Medicine, Jawaharlal Nehru Medical College, Datta Meghe Institute of Medical Sciences, Wardha, IND; 2 Otolaryngology - Head and Neck Surgery and Surgical Oncology, Jawaharlal Nehru Medical College, Datta Meghe Institute of Medical Sciences, Wardha, IND

**Keywords:** headache, migraine, aura, lifestyle, triptans, history taking, genetic predisposition, episodic, neurological disease

## Abstract

The second most prevalent cause of years of disability worldwide is migraine, a neurological condition that causes a persistent headache that is lifelong. This condition affects more than a billion people globally. Its widespread prevalence and concomitant impairment have several adverse effects. Migraines are brought on by numerous behavioural, ecological, and genetic factors. There are various types of migraines, of which migraine without aura is the most common. The objective of this article is to determine the causes of migraine headaches, review the appropriate diagnosis of migraine, and describe the migraine headache management alternatives that are available. Various treatments for migraine are available on the market. Among the many types of headaches, migraines are neurological in nature and are inherited in some people. It has four phases: prodromal phase, aura, attack phase, and postdrome phase. Stress, anxiety, changes in the female endocrine system, bright lights, loud noises, foul odour, excessive or insufficient sleep, changes in the environment or the climate, flashes of light or intense lighting, overexertion, missing meals, drinking alcohol and smoking, quitting caffeine, and taking too many migraine drugs are some of the triggering factors for migraine. Diagnosis of migraine mainly relies on a good history. Appropriate prevention can be done for specific indications. Treatment mainly revolves around medications like analgesics, triptans, ergot derivatives, and newly derived biologics. Lifestyle modifications are also essential as many daily life factors contribute to migraine. Migraine can be well-managed if sufficient attention and care are given along with proper medications and guidance. The patient should not ignore symptoms and report to the physician at the earliest so that correct management can be done.

## Introduction and background

Migraine is a neurological illness that is a type of primary headache that is persistent and lifelong and is one of the causes of years of disability worldwide [1]. Over a billion individuals are impacted by this illness worldwide. Its extensive incidence and accompanying impairments have a variety of detrimental and significant impacts on not only those who are directly affected, but also on their families, friends, co-workers, employers, and the community as a whole [2]. Migraine is not just a simple headache, it is a complicated condition with genetic influences that manifests as periods of moderate to severe headache, most frequently unilateral, and often accompanied by nausea, photophobia, and phonophobia. A migraine episode is a complex neurovascular event that can last from hours to days [3].

History

The word migraine was eventually derived from the term "hemikrania" (half-head) used by Galen of Pergamon, then the word underwent some changes going through Latin, then French, and was fixed on "migraine". The Ebers Papyrus has an early description of migraine that dates to around 1500 BC in ancient Egypt. Writings of the Hippocratic School of Medicine from 200 BC documented the visual aura that may precede the headache and a partial solace from vomiting [4]. Louis Hyacinthe Thomas established the distinction between migraine in the presence of an aura (ophthalmic migraine) and migraine in the absence of aura in 1887 (migraine vulgaire) [5]. William Harvey suggested trepanation as a migraine cure in the 17th century. While various migraine treatments were attempted, in 1868, a material known as fungus ergot was used to extract ergotamine for the treatment of migraine [6].

Problem statement

About 91% of men and 96% of women encounter headaches, of which approximately 6% of men and 18% of women experience migraine (one-year prevalence). People in their 20s and 30s and socioeconomically disadvantaged categories are those where migraine is most prevalent. It is linked to a rise in the frequency of both anxiety symptoms and distress [7].

Inheritance

Several behavioural, ecological, and genetic factors contribute to migraine, but it has been discovered that abnormalities in some genes are linked to the onset of migraines. There are probably different effects of these genes on different people. More than half of those who suffer from migraine have at least one family member who also has the condition but there is no apparent pattern of inheritance [8]. An uncommon monogenic migraine with aura (MA) subtype known as hemiplegic migraine is caused by mutations in the calcium voltage-gated channel subunit alpha 1A (CACNA1A), ATPase Na+/K+ transporting subunit alpha 2 (ATP1A2), and sodium voltage-gated channel alpha subunit 1 (SCN1A) genes, all of which encode for ion channel and transport proteins [9].

Types of migraine

Migraine is a type of primary headache with two main types, the first of which is migraine with aura, formerly known as classical migraine, in which sensory and other neurological symptoms precede a migraine attack [10]. The second type is migraine without aura, formerly known as common migraine. It is the most common type. One of the subtypes is a chronic migraine, also known as high-frequency episodic migraine, which involves 15 headache days each month, with eight of those days featuring migraine-like symptoms and lasting longer than three months [11].

Other rare types include migraine aura without headache (silent migraine), abdominal migraine, hemiplegic migraine, ocular migraine, status migrainosus, etc. [12]. Status migrainosus refers to a migraine that persists for more than three days. Symptoms are dazzling light sensation or other visual alterations (aura), nausea and vomiting, and an inability to think clearly. As the condition lasts for three days, the patient may become dehydrated due to excessive vomiting. The patient can also develop insomnia due to severe pain [13]. Migraine headache accompanied by hemiplegia causes a rare condition known as hemiplegic migraine. People who are impacted are said to have a migraine with aura. Two types of hemiplegic migraine are hereditary and sporadic [14].

The distinction between migraine and tension headache

Those who suffer from tension headaches describe an uncomfortable band across their forehead or pressure on either side of their head. Although painful, the discomfort is not as bad as a migraine. Contrarily, a migraine typically causes more pain on one side of the head. In addition, the patient might see brilliant lines or dots in their range of vision, along with sensitivity to light, or an aura, as shown in Table 1 [15].

**Table 1:** Difference between migraine and tension headache Adapted from [16].

Characteristics	Migraine	Tension headache
Intensity	Moderate to severe	Mild to moderate
Headache location	Often unilaterally	Always bilateral
Headache duration	4-72 hours	30 minutes to 1 day
Headache frequency	Recurrent	Infrequent
Demographics	Affects women 2-3 times more	Higher prevalence in women than man
Symptoms	Aura, severe headache, sensory difficulties	Pericranial headache, no sensory symptoms

## Review

Migraine is a genetically influenced neurological condition. Most people frequently imagine a severe headache when they hear the term "migraine", but migraine can have various symptoms, and headache is just one of them [17]. The International Headache Society has given diagnostic criteria for migraine with aura, as given in Table 2, and migraine without aura, as given in Table 3.

**Table 2:** Diagnostic criteria for migraine with aura by the International Headache Society Adapted from [18].

Diagnostic criteria for migraine with aura by the International Headache Society	
(A)	Minimum two attacks meeting B-D criteria.
(B)	Having at least one of the following characteristics but not motor weakness.
1.	Visual symptoms that are completely reversible and may have either good characteristics (such as dots, lines, or flashing lights) or negative characteristics (visual loss).
2.	Completely reversible sensory sensations with either positive (pins and needles) or negative (numbness) aspects.
3.	Completely reversible dysphasia.
(C)	Minimum two of the following:
1.	Homonymous ocular symptoms and/or unilateral sensory symptoms.
2.	A minimum of one aura symptom gradually appears over a period of five minutes, or a series of various aura symptoms appear over five minutes.
3.	Each symptom lasts between five minutes and one hour.
(D)	Headache that meets the B-D criteria for migraine without aura, begins during the aura or within one hour after the aura.
(E)	Not linked to any other condition.

**Table 3:** Diagnostic criteria for migraine without aura by the International Headache Society Adapted from [19].

Diagnostic criteria for migraine without aura by the International Headache Society.	
(A)	Minimum two attacks meeting B-D criteria.
(B)	Headache lasting from 4 to 72 hours (untreated or unsuccessfully treated).
(C)	Minimum two of the following features:
1.	Unilateral headache
2.	Throbbing in character
3.	Moderate to severe in intensity
4.	Aggravated by avoiding physical activity
(D)	Minimum one of the following during headache:
1.	Nausea and/or vomiting
2.	Photophobia and phonophobia
(E)	Not linked to any other condition

Phases of migraine

**Figure 1:**
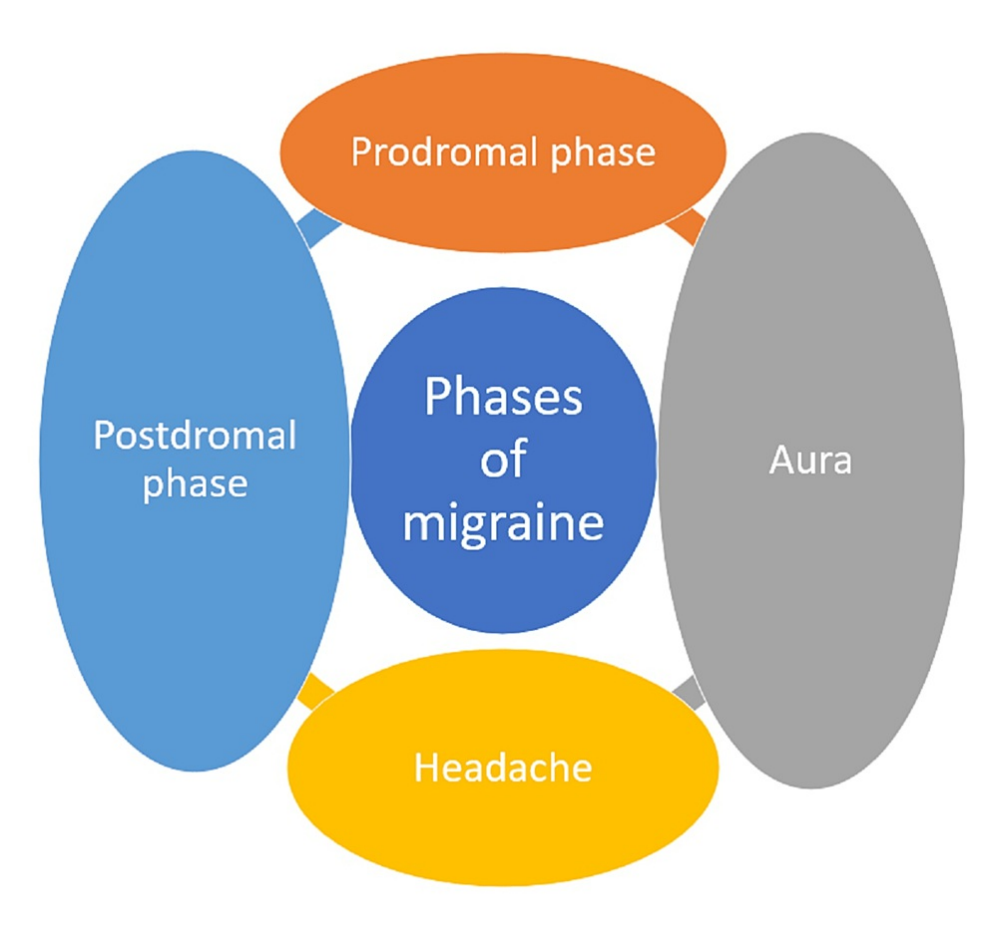
Phases of migraine

A typical migraine attack may consist of four phases, lasting as low as six hours to a whopping 72 hours. Some of these phases are not seen in all migraine patients. The phases are shown in Figure 1 [20].

Prodromal Phase

Hours or days prior to the headache, some non-painful symptoms start to appear, which include yawning, mood swings, trouble focusing, neck pain, exhaustion, thirst, and an increased frequency of urination [21].

Aura

Also referred to as the "warning stage", it can eventually appear before the commencement of a migraine headache. Most often, aura affects a patient's vision. Some people have experienced confusion, disorientation, and dizziness [22]. Sensory disruptions, such as spots, flashes, and zigzags in the visual field, are referred to as migraine aura. Some persons complain of tinnitus, vertigo, or even difficulty in speaking clearly. This condition can appear 30 to 60 minutes before a migraine attack or even during it. It is essential to understand that not all migraine attacks include an aura [23].

Headache

Also known as the attack phase, it can persist for hours. The patient wishes to rest quietly during this time and finds it challenging to carry out their daily activities. The pain usually starts in the supraorbital region, particularly around the eyebrows, which then radiates to the temporal area on one side of the head. Some patients also complain of pain in the cervical region in the neck [24]. Pulsating or throbbing pain is characteristic of migraine. The pain aggravates on exposure to lousy odour, bright light, loud sounds, stress, and gastrointestinal problems like constipation [25].

Postdrome Phase

This phase is also referred to as the post-migraine phase. This latter stage of a migraine is frequently compared by migraine patients to a hangover from alcohol intoxication. It could be as terrible as a migraine attack itself. The patient feels worn out, exhausted, and drained. The patient complains of neck pain, trouble focusing, intestinal problems, mild headache, feeling dehydrated, and mood swings [26].

Triggering factors for migraine include stress, anxiety, female's endocrine fluctuations, a flash of light or intense lighting, loud sounds, unpleasant odour, extra or insufficient sleep, fluctuations in the surroundings or the climate, overexertion (too much physical activity), caffeine withdrawal, smoking and drinking alcohol, missing meals, and excess of medications for migraine. Food triggers are reported by 12-60% of patients [27]. Numerous reports show that tyramine, which is found naturally in foods like chocolate, liquors, cheese products, processed meat, and other foodstuffs, can trigger migraine in some people. Similarly, monosodium glutamate (MSG) is widely cited as a migraine trigger [28].

Pathophysiology

It is believed that several factors influence the onset and intensity of migraines.

Meningeal Nociceptor Activation

Activation of meningeal nociceptors and dilation of intracranial blood vessels: With trigeminal neuronal activation, there is a release of neuropeptides (vasoactive) such as substance P or calcitonin gene-related peptide (CGRP), which leads to vasodilatation. The release of inﬂammatory molecules, such as histamine, bradykinin, 5-hydroxytryptamine (5-HT), and prostaglandins (PGs), neurogenic inflammation, and the activation of meningeal nociceptive receptors are all contributors to the activation of the trigeminovascular pathway [29]. Changes in cortical enzymatic activity (pro-inflammatory chemicals), neurogenic inflammation, excitation and inhibition of the cerebral cortex, and cortical spreading depression lead to activation of meningeal nociceptors and neurogenic inflammation [30].

Genetic Predisposition

In those with migraine, the brain cannot acclimate to external stimuli (such as stress or hormonal changes), which leads to an overexcited brain [31]. External physiological and emotional stimulation (such as hormonal changes and stress) activates the autonomic nervous system. The hypothalamus responds by altering homeostasis. Hypothalamic neurons then influence the autonomic nervous system, causing it to shift towards a parasympathetic tone. Finally, intracranial blood vessels, particularly those in the meninges, constrict and dilate [32]. The theory of vasodilation is now considered as obsolete in the causation of migraine. A neuromodulator called adenosine might be implicated. Adenosine is a chemical released when adenosine triphosphate (ATP) is gradually broken down. It works by relaxing blood vessels and lowering heart rate to put the body and brain into a low-activity state, which is expected just before and during the first stages of sleep. It has been discovered that adenosine levels are elevated during migraine attacks [33]. The ability of caffeine to inhibit adenosine may account for its ability to lessen migraine symptoms. Serotonin is a neurotransmitter that is thought to be implicated in low levels. Since levels of CGRP increase during a migraine attack, it has been discovered that they contribute to the pathophysiology of the pain resulting from them [34].

Complications of migraine include status migrainosus, migrainous infarction, persistent aura without infarction, and seizures triggered by migraine; other complications include depression, anxiety, insomnia, etc. [35].

Diagnosis

Diagnosis of migraine headache is mainly clinical. A detailed history must be taken along with a proper central nervous system examination. The patient must fully describe all the symptoms. The history plays a vital role in diagnosing migraine, and the examination's primary goal is to find any additional issues that might aggravate a pre-existing propensity for migraine [36]. If the right questions are asked, history is always enough for diagnosing migraine headaches. The patient usually presents with a history of unilateral headache, which is pulsatile in character, beginning in the supraorbital region and progressing to the temporal area, associated with nausea or vomiting along with irritation from loud sounds or exposure to bright light. The patient always says they have these symptoms quite frequently for more than two or three times a week (if a chronic case) [37]. Due to the failure of many earlier treatments, patients are frequently discouraged and even frustrated. Cure for headaches often fails due to a routine, hastily obtained, uninteresting history. One of the crucial conditions is time. The physician must be sympathetic and comprehend the patient's suffering. Most patients have undergone numerously hurried and unsuccessful consultations, after which the headache has not subsided, and they have not been informed of the cause [38]. Start by asking questions regarding the pain pattern, such as when and how the headaches start, if they are continuous or episodic, or as is frequently the case with chronic migraine (constant with periodic worsening), the length of bouts or exacerbations, and whether any triggers or aggravating factors exist [39]. Ask about the nature of the pain, including its location, character, and severity. It is also essential to determine whether there are any accompanying symptoms, such as nausea, sensitivity to light, sound, smell, touch, or movement, and symptoms that precede or accompany attacks, such as excessive tiredness or energy, yawning, excessive urination, neck pain, vertigo, or visual or sensory disturbances [40]. Ask about any symptoms that could point to different primary or secondary headache diseases, such as eye-watering, conjunctival infection, nasal congestion, ptosis, eyelid oedema, perspiration, anxiety, fever, neck pain, and rash [41]. The utilization of past and present treatments, as well as the timing of their administration, is essential to note. Patients should be asked to bring a list of all previously attempted medications and dosage information and be questioned; they should be asked why these therapies were discontinued [42]. Ask about the patient's medical history, current non-headache drugs, allergies, family history (particularly of headaches), social history, and inquiries concerning depression, anxiety, and sleep disturbances (including occupation, smoking status, and levels of alcohol and caffeine consumption). Asking about migraine symptoms like frequent stomach aches, motion sickness, or a more than usual predisposition to hangovers can also be beneficial [43].

Management

Medications used to relieve migraine pain should be taken when the first sign of oncoming migraine appears (aura). Medications that can be used for mild cases are paracetamol, nonsteroidal anti-inflammatory drugs (NSAIDs) (naproxen 500 mg twice a day), ibuprofen 200-400 mg given four hourly, along with antiemetics for treating nausea and vomiting. For severe cases, triptans can be used, which include 5-HT receptor agonists like rizatriptan and eletriptan taken orally only. Sumatriptan (taken orally/nasally/subcutaneously) and zolmitriptan (taken orally/nasally) are also effective. Ergot alkaloids can also be used like dihydroergotamine (headache recurrence is least for ergotamine) [44]. Some of the issues with triptans are that they are ineffective in classical migraine. It is contraindicated in patients with coronary artery diseases and stroke [45]. Newer treatment modalities include monoclonal antibodies against CGRP, which include drugs like erenumab, galcanezumab, and eptinezumab [46]. A newly found drug lasmiditan is a 5-hydroxytryptamine receptor 1F (5-HT1F) agonist developed to meet the unmet needs of patients having cardiovascular risk factors and diseases. It is also effective in patients who do not respond well to primitive treatment modalities available on the market [47]. Lifestyle modifications also significantly prevent migraine, including adequate sleep, less stress, proper water intake, good diet, proper exercise, avoidance of smoking and alcohol, decreased dependence on caffeine, and no abuse of analgesics [48].

Prophylaxis

There may be a lack of utilization of migraine prevention. This may be due to patients' reluctance to treat due to undesirable side effects, but also because some physicians are inexperienced with preventive medications. A referral may be indicated when migraine control is inadequate and knowledge of migraine prevention is required. However, managing prevention in general practice is frequently appropriate. When it works, it has a significant positive impact on a patient's quality of life [49]. First-line drugs include propranolol, amitriptyline, and topiramate. Second-line drugs include telmisartan, valproate, and venlafaxine. Third-line drugs include clonidine, flunarizine (calcium channel blocker), and pizotifen [50]. Other treatment modalities include onabotulinum toxin A, greater occipital nerve block, and supraorbital transcutaneous stimulation. Indications for migraine prophylaxis are as follows: if more than two migraine bouts occur annually, if the average attack lasts more than 24 hours, or if the headaches severely disrupt the patient's lifestyle, leaving them severely disabled for three or more days [51].

Differential diagnoses include sinusitis headache, tension-type headache, stroke, aneurysm in the brain, tumour in the brain, epilepsy, glaucoma, and meningitis [52].

## Conclusions

Migraine is a complex neurological disorder often under-estimated, under-recognized, under-diagnosed, and under-treated. Patients across the globe kept on suffering due to migraine for years and did not get proper attention, and the severity of the disease is underestimated by physicians. Many treatment options are available in the market, but the patients are not getting a permanent cure. This happens due to a lack of knowledge about the disease and treatment options available among inexperienced physicians and ordinary people. People tend to abuse painkillers whenever they experience headaches and ignore the possibility of getting migraine. Due to this painkiller abuse, migraine worsens, and the patient suffers from gastrointestinal symptoms. There is much less awareness about the disease in many rural areas too. Migraine can be considered a silent pandemic as it is prevalent throughout the globe, and the incidence keeps increasing daily. Among all types of headaches, migraine accounts for 6% of cases in men and 18% in women, which is alarming. For proper diagnosis of migraine, a physician must know the art of history taking because history is the key to the diagnosis of migraine. One can diagnose the disease only by history, and no expensive investigations are required. The main objective behind writing this article is to make people aware of migraine and its symptoms, complications, prevention, and various treatment options available on the market.
